# The Microscope in Medicine

**Published:** 1891-02-28

**Authors:** Frank J. Wethered


### The Microscope in Medicine.

By Frank J. Wethered, M.D.

### VII.—METHODS OF STAINING SECTIONS OF THE NERVOUS SYSTEM.

It has already been hinted in the previous articles, that in order to procure sections of the central nervous system special methods have to be adopted besides those used for the preparation of ordinary specimens. This is on account of the complex structure of the nervous system. Sections of the spinal cord may indeed be stained by the processes described in Article V., but their component parts will not be satisfactorily differentiated. Within the last few years certain methods dependant upon chemical reactions have been introduced, by means of which the most minute nerve fibres stand clearly out from the neuroglia, producing the most elaborate pictures, distinct in every detail. Those methods, which it is proposed to describe in this article, are those introduced by Weigert and Pal, and the process of staining by aniline blue-black. As sections of the brain and spinal cord are stained in a similar manner, and it is the latter of which an examination is most frequently required, the present description will be applied to the spinal cord. The cord is most safely removed without injury from the front. It is at once placed in Müller's Fluid contained in a tall glass jar, and the liquid should be changed several times as described in Article III. When sufficiently hardened, as estimated by the touch, it is washed in water, and kept in 80 per cent. alcohol until an opportunity is found for cutting it. The spinal cord is then divided into a number of transverse segments about a quarter of an inch in thickness, which are then transferred to equal parts of alcohol and ether preparatory to embedding in celloidin. Sections are then cut in the usual manner, but one fact must be carefully borne in mind, and that is that the specimens being extremely friable they must be manipulated with great caution. If it be desired to cut a series of sections, it is advisable to embed the cord in paraffin instead of celloidin, and to use the Cambridge rocking microtome.

Weigert's Method of Staining.—The first step consists in placing the sections in a solution of acetate of copper, made according to the following directions: A saturated solution of acetate of copper, 1 part; distilled water, 1 part. After remaining in this preparation for twenty-four hours, they are removed to a capsule of water for twelve hours, the water being changed two or three times. The next stage is immersion in Weigert's hæmatoxylin: Hæmatoxylin crystals, 1 part; alcohol (90 per cent.), 10 parts; concentrated solution of lithium carbonate, 1 part; distilled water, 90 parts. This solution is not ready for use for at least forty-eight hours after it has been made. The sections must remain in it for one or two days, and are then transferred to water. They should now have a deep black colour, and be almost opaque. If such be not the case they must be replaced in the hæmatoxylin, or a few drops of a solution of carbonate of lithium may be added to the water when very often the desired result is obtained. The water must be frequently changed until the washings are no longer coloured. This process is a very important one and must be thoroughly carried out, for if any excess of stain be left in the sections they will appear mottled. They have now to be "differentiated." The solution for this purpose has the following composition: Ferricyanide of potassium, 2·5 parts; borax, 2 parts; water, 200 parts. Almost immediately on being placed in this solution the sections will be noticed to undergo a remarkable change. They will become much lighter in colour, and the opacity rapidly gives place to transparency. They must now be carefully watched until the white and grey substances are clearly defined. The time occupied until this is completed varies, being seldom under five minutes or over half an hour. If allowed to remain too long, however, they become of a unform brownish colour, and are then entirely spoilt. When ready, they should be removed to water, and left for about half an hour; they are then dehydrated, cleared in creosote, and mounted in balsam. One caution may here be given; celloidin is soluble in absolute alcohol, and when this support is removed sections are very apt to fly to pieces. It is therefore advisable to dehydrate in 95 per cent. alcohol, allowing a little extra time for the process. Stained as described above, the white substance of the nerve fibres appears coloured a purple-black, whilst the axis-cylinders, ganglion cells, and connective tissues, take on an orange-brown colour. If there are any degenerated tracts, as in cases of sclerosis, these are also clearly picked out in the latter colour.

Pal's Method.—This is somewhat similar to the foregoing, but the results are better; it takes a much shorter time, the nerve fibres are more sharply defined, and the remaining parts being colourless, a counter-stain may be employed to demonstrate the nuclei of the connective tissue and the ganglion cells. The cord is hardened and cut as we have just described, and the sections, when ready, are placed in the following solution: Hæmatoxylin, ·75 parts; distilled water, 90 parts; alcohol, 10 parts. Immediately before use a few drops of a saturated solution of lithium carbonate are added in the proportion of three drops of the solution to 10 c.c. of the hæmatoxylin. After remaining in this stain for five or six hours the sections should have assumed the dark blueish-black colour described under Wiegert's process. They are then washed in water until all the excessive stain is removed. The differentiation is conducted in two stages. The sections are first placed in a 25 per cent. solution of permanganate of potash. In this they remain from fifteen to twenty seconds, and are then removed to "Pal's solution"—Potassium sulphite, 1 part; oxalic acid, 1 part; distilled water, 200 parts. Whilst in this liquid the sections must be carefully watched for the distinct separation of the grey and white matters. This is usually completed in one or two minutes. Occasionally black spots appear, and then the specimens must be replaced for a few seconds in the permanganate of potash, and again passed through Pal's solution to water, where they should remain for a quarter of an hour. The ganglion cells nuclei, and axis, cylinders may now be stained in alum carmine: Carmine, 1 part; alum (five per cent. solution), 100 parts. This liquid is exposed to the air for twenty-four hours and then filtered. Ten minutes' immersion in this stain is sufficient, and the specimens are then washed in water, dehydrated in 95 per cent. alcohol, cleared in creosote, and mounted in balsam. Another modification of the above processes is known as the "Pal-Exner Method." In this the spinal cord, freshly removed, is at once placed in ten times its bulk of a half per cent. solution of osmic acid. It is allowed to remain two days, and is then washed in water. Segments are then cut and dipped for a few seconds into absolute alcohol, after which they are embedded and cut. They are differentiated in permanganate of potash or Pal's solution in the manner described above, and are then finished in the usual way.

Aniline Blue-black.—This is a very rapid and simple method, and is particularly applicable to unhardened specimens of brain. It is described by Lewis as follows ("Manual of Brain Examination"): "Sections sufficiently thin, cut by an ether-freezing microtome, are floated on to water, from which they are transferred for a few minutes to a two per cent. solution of osmic acid, for the purpose of hardening. They are afterwards washed in water, and then immersed in a quarter per cent. solution of aniline blue-black for a couple of hours. After again washing in water, each section is arranged on a slide and allowed to become quite dry, after which it is mounted by the addition of a drop of Canada Balsam and a cover glass." The section will now be stained a slaty-blue colour, the nerve-cells being tinged darker.

